# When Mechanics Rewrites Chemistry: Divergent Pathways of Hardening and Softening in High Strength Al‐Alloys

**DOI:** 10.1002/advs.77786

**Published:** 2026-09-16

**Authors:** Huan Zhao, Linze Li, Shaolou Wei, Jueyi Qi, Jiewen Xiao, Degang Xie, Xie Zhang, Gang Liu, Dierk Raabe, Jun Sun

**Affiliations:** ^1^ State Key Laboratory for Mechanical Behavior of Materials Xi'an Jiaotong University Xi'an People's Republic of China; ^2^ Max‐Planck‐Institute for Sustainable Materials Düsseldorf Germany; ^3^ School of Materials Science and Engineering Northwestern Polytechnical University Xi'an People's Republic of China

**Keywords:** Al alloys, dislocation dynamics, mechano‐chemical coupling, solute clustering, precipitate dissolution

## Abstract

High‐strength 7xxx Al alloys are the backbone of lightweight structural engineering, yet their broader application is hindered by limited ductility. This deficiency marks a breakdown of traditional strengthening paradigms where microstructure features designed for peak strength trigger localized softening, creating a fundamental mechanism trade‐off. Here, we reveal that plastic flow activates two divergent mechano‐chemical pathways during deformation, namely, deformation‐driven hardening and shear‐induced softening that govern strain localization and ductility. In the solution‐treated state, strain‐induced nanoscale solute clustering promotes homogeneous dislocation storage and sustained work hardening. Conversely, in the peak‐aged state, localized shear drives precipitate dissolution, creating precipitate‐free channels. These soft channels not only amplify strain localization but also act as preferential sites for the ingress of environmental species, which further weakens the lattice and accelerates catastrophic failure. Leveraging these findings, we show that mechanical response can be engineered by balancing homogeneous plastic flow against shear‐driven local chemical phase dissolution and the associated soft channels. This study provides critical mechanistic insights into the dynamic mechanochemical response of high‐strength Al alloys, offering physical guidelines for future microstructural tailoring to suppress severe deformation localization.

## Graphical abstract

Mechanics reshapes chemistry: plastic flow actively governs two divergent, atomic‐scale mechanochemical pathways that tailor strain localization and ductility in high‐strength 7xxx Al alloys. Rather than relying on static structures, deformation dynamically reinforces the solution‐treated state via clustering, yet destabilizes the peak‐aged condition through shear‐driven precipitate dissolution. This dissolution creates severely damaged channels, accelerating environmental ingress and subsequent catastrophic fracture.

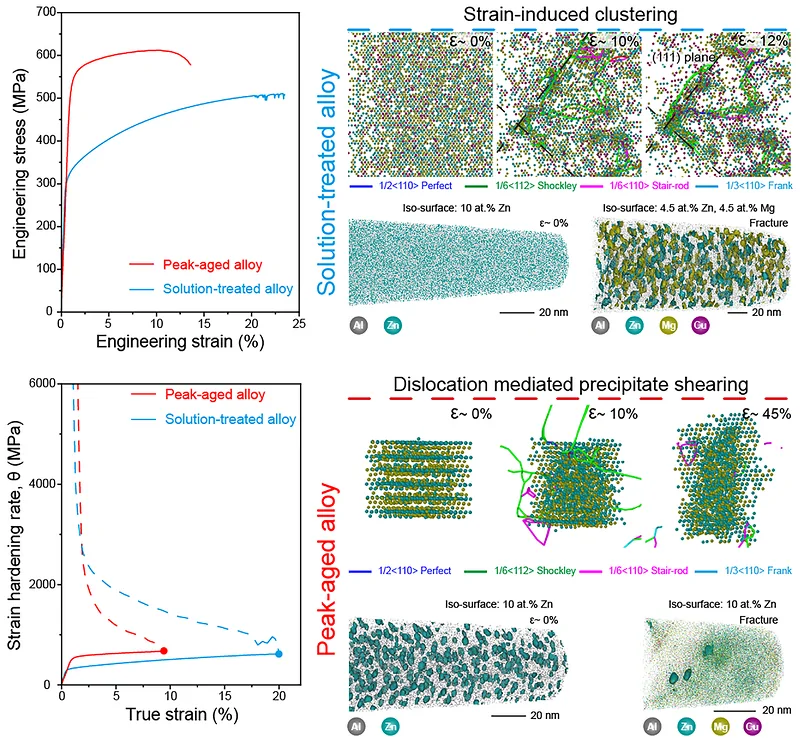

## 1 Introduction

High‐strength Al–Zn–Mg–Cu (7xxx) alloys are essential structural materials in aerospace and transportation, where superior strength‐to‐weight ratios with yield strengths exceeding 500–600 MPa are required [1, 2, 3]. Their mechanical performance is governed by microstructure, which is tailored through thermomechanical processing and aging [4]. While peak‐aging achieves superior strength through a dense dispersion of (Mg, Zn)‐rich precipitates [5, 6], it also causes a loss in ductility and susceptibility to failure. In contrast, solution‐treated alloys exhibit lower strength but offer significantly enhanced strain hardening, formability, and damage tolerance [7]. This stark contrast reveals the fundamental challenge of the strength‐ductility trade‐off in 7xxx alloys [8, 9], a problem that has long hindered the design of high‐performance structural parts due to the requirement for a compromise between high load‐bearing capacity and structural reliability.

When straining and precipitation occur together, their dynamic interaction governs the mechanical response through complex dislocation‐chemical transformation pathways [10]. This creates a divergence in the mechanical response of these essential lightweight alloys, with either high peak strength causing low damage tolerance, or higher solid solution leveraging better strain hardening and damage tolerance. An untapped synergetic regime for alloy and process design lies between these extremes, in which solutes and precipitates interact with gliding dislocations in a way to reconcile both advantages, a state that can be triggered by deformation [11, 12, 13, 14]. While the two extreme initial microstructure states have been well‐studied [5, 15], the dynamic nature of the deformation processes leading to either stable plastic flow or early failure remains a fundamental trade‐off challenge. In particular, it is unclear how strain‐induced structural and chemical evolution creates the local instabilities that induce intense strain localization and the associated softening [16]. This phenomenon triggers a critical breakdown in ductility, because the localization of plastic strain into narrow bands leads to soft channels and early failure [10, 16, 17]. Understanding the specific pathways during deformation by which the dynamic interactions govern such instability is crucial for bridging the mechanistic gap between microstructural evolution and failure. Improving our understanding of the fundamental reasons behind this localization process is thus essential to reveal the origin of ductility loss and provide physical guidelines for suppressing severe deformation localization in high‐strength structural alloys. While conventional microstructural control focuses primarily on the initial static microstructure, plastic flow dynamically reshapes local chemistry. Clarifying such stress‐driven chemical evolution is critical to advancing processing‐structure‐property strategies, providing fundamental insights into deformation dynamics.

To do so, we use a multi‐scale approach integrating in situ synchrotron x‐ray diffraction, in situ scanning electron microscopy (SEM), atomic force microscopy (AFM), transmission electron microscopy (TEM), atom probe tomography (APT), and atomistic simulations. This allows us to probe the dynamic interplay between dislocation motion and the local chemical redistribution in Al–Zn–Mg–Cu alloys. By directly comparing solution‐treated and peak‐aged states, we elucidate how distinct initial microstructural states and their change upon dislocation shear govern the deformation behavior through the evolving interactions with dislocations. These findings provide new insights into the mechano‐chemical coupling during deformation, elucidating the mechanistic connection between dynamic microstructure evolution of alloys and their macroscopic resistance to localized failure.

## 2 Results

**FIGURE 1:**
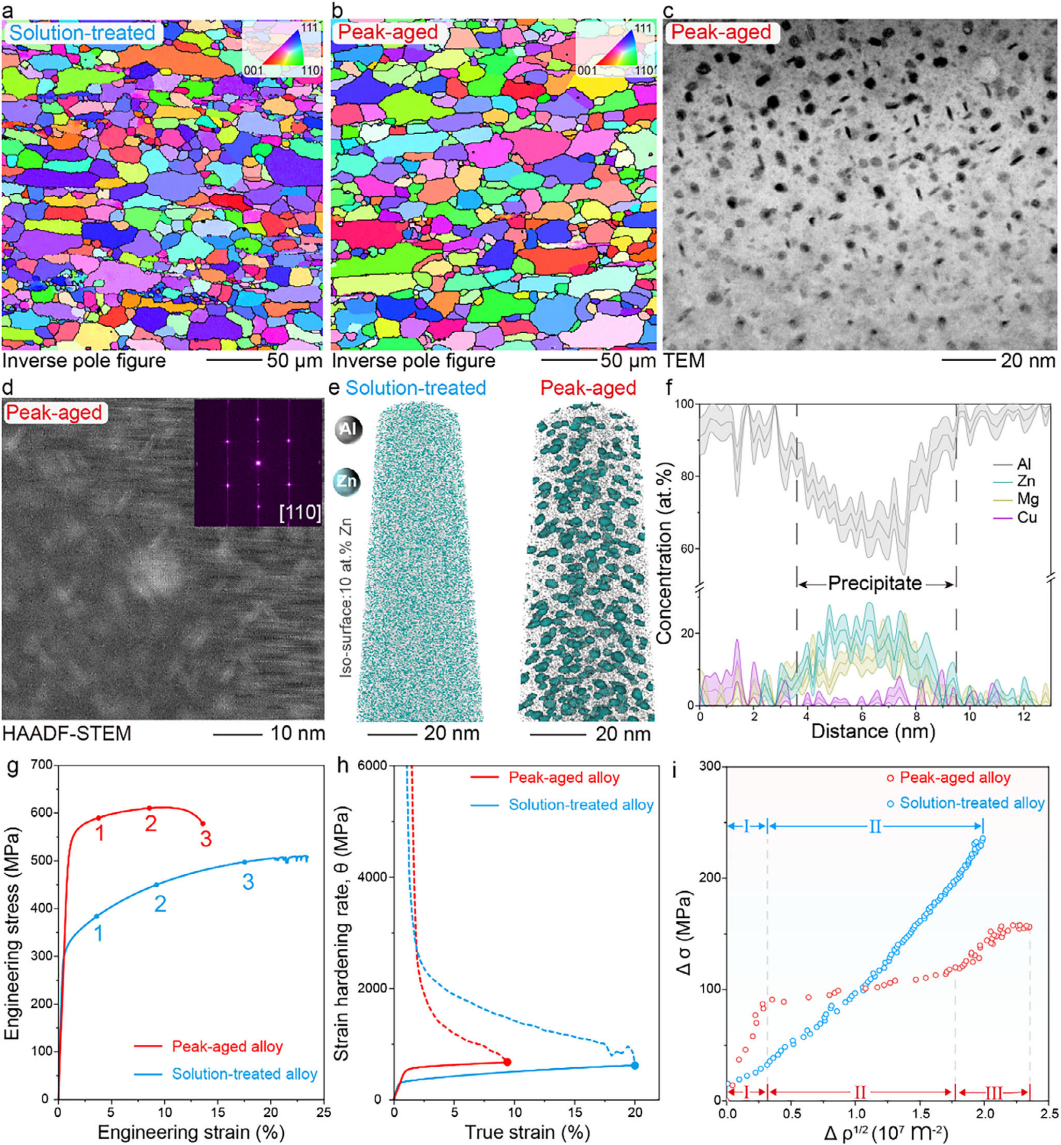
Initial microstructural characterization, mechanical response, and dislocation density evolution of the Al–Zn–Mg–Cu alloys. (a, b) EBSD ‐ IPF maps along the tensile direction of the solution‐treated and peak‐aged states, respectively, showing equiaxed grains. (c) TEM and (d, **inset**) HAADF‐STEM images of the peak‐aged alloy resolving a high number density (10^24^ m^−3^) of precipitates. (e) APT reconstructions of the solution‐treated state (left) exhibiting a supersaturated solid solution and the peak‐aged state (right) showing solute‐enriched precipitates. (f) 1‐D concentration profile for the peak‐aged precipitates in (e right), showing Zn and Mg. (g) Engineering stress–strain curves obtained from uniaxial tensile tests at a strain rate of 1.0 × 10^−3^s^−1^. (h) Corresponding true stress–strain curves with strain hardening rate (θ) plots for both microstructural states. (i) Evolution of strength increment (Δσ) as a function of the square root of dislocation density (Δρ^1/2^) measured by in situ high‐energy synchrotron x‐ray diffraction (HEXRD).

The initial microstructures of the Al–Zn–Mg–Cu alloy in the solution‐treated and peak‐aged states are shown in Figure 1a–f. Electron backscatter diffraction (EBSD) inverse pole figure (IPF) maps (Figure 1a,b) reveal that the alloy has comparable grain structures in both conditions, featuring fully recrystallized grains with random orientations. The peak‐aged microstructure consists of a high number density (10^24^ m^−3^) of uniformly dispersed GP zones and η’ precipitates with an average size of 5 nm [5], as confirmed by TEM, high‐angle annular dark‐field scanning transmission electron microscopy (HAADF‐STEM)(Figure 1c,d), and APT analysis (Figure 1e, right). The proximity histogram (Figure 1f) further quantifies the solute enrichment, revealing a concentration of approximately 50 at.% (Zn+Mg) in the precipitate core. APT reconstruction of the solution‐treated state (Figure 1e, left) confirms a nearly homogeneous supersaturated solid solution.

The tensile behavior of the alloy is controlled by these initial microstructural states, as shown in the engineering and true stress–strain curves acquired at a strain rate of 1 × 10^−3^ s^−1^ (Figure 1g,h). The solution‐treated alloy exhibits a yield strength of 320 MPa and a uniform elongation of 24%, featuring a substantial strain‐hardening of 190 MPa that reaches an ultimate tensile strength of 510 MPa. In contrast, peak‐aging increases the yield strength to roughly 570 MPa and the ultimate tensile strength to around 620 MPa, but at the cost of massive ductility loss, to only 14%. The negligible increase in flow stress beyond yielding in the peak‐aged alloy, compared to the sustained strain hardening of the solution‐treated state, signifies a severely compromised strain‐hardening capacity and diminished plastic stability during monotonic loading.

The work‐hardening rate (ϴ) as a function of true strain in Figure 1h further elucidates the different deformation behavior for the material in its two different microstructural states. While both variants exhibit an initial drop in hardening upon yielding, the decay kinetics are very different. The solution‐treated alloy exhibits a steady and gradual decline, with its hardening capacity still persisting at a true strain exceeding 20%. Throughout the post‐yield regime, the work hardening rate of the solution‐treated alloy reaches up to 120% higher than that of the peak‐aged state, maintaining a maximum hardening advantage of 800 MPa. As illustrated by the crossover points in Figure 1h, the solution‐treated alloy fulfills the Considère criterion [18] (ϴ = σ) at a high strain of 20%, thus achieving a uniform elongation of 24%. In contrast, the peak‐aged alloy displays a steep decline in its work hardening rate within the strain range of 2–5%, reaching the crossover point at a strain of 9% and leading to a limited ductility of 14%.

The microscopic reason for these hardening characteristics is elucidated by the evolution of the dislocation density, quantified via the Williamson‐Hall method [19] of the x‐ray diffraction data acquired in situ during tensile deformation. The relationship between the strength increment (Δσ) and the square root of dislocation density (Δρ^1/2^) is presented in Figure 1i. While the alloys in both microstructure states start deformation with similarly low initial dislocation densities of about 2 × 10^14^ m^−2^ (Figure S1), their accumulation kinetics diverge upon loading. The solution‐treated alloy follows a sustained linear Taylor hardening trend, indicative of stable forest dislocation storage and robust strain‐hardening capacity. To evaluate this response, we calculated an effective fitting coefficient α using the Taylor equation [20] (see Methods). In Stage I, the solution‐treated alloy exhibits an α value of 0.2, which increases to 0.37 in Stage II. This monotonic linear increase in α value suggests that while the plastic flow remains homogeneous, the underlying deformation resistance is primarily governed by dislocation forest storage, providing the structural basis for the alloy's persistent hardening response. In contrast, the peak‐aged alloy exhibits a non‐linear deviation with a clear inflection point at 5% strain. During Stage I, it exhibits an exceptionally high initial hardening rate with an α value of 0.92, likely driven by intense interaction between dislocations and the precipitates. However, beyond a critical strain of 5%, the α value drops to a negligible value of 0.06. Although a slight recovery to a value of 0.23 is observed in Stage III, the overall trend captures the breakdown of strain‐hardening capacity.

**FIGURE 2:**
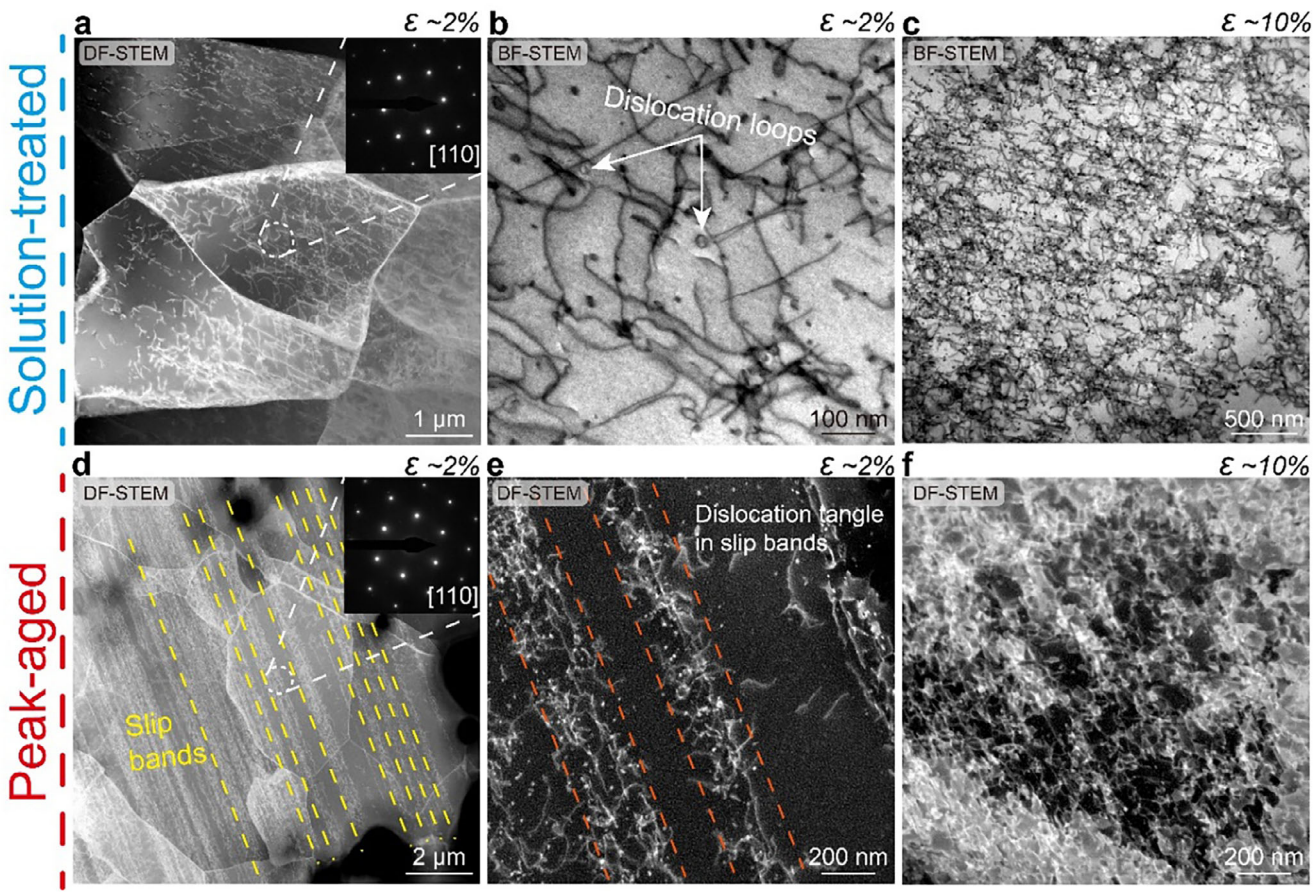
Evolution of dislocation substructures and deformation localization in solution‐treated and peak‐aged alloys. (a–c), TEM micrographs of the solution‐ treated alloy. Inset in a shows the corresponding selected area electron diffraction (SAED) pattern along the [110] zone axis. (d–f) TEM observations of the peak‐aged alloy. Inset in d shows the corresponding SAED pattern along the [110] zone axis.

To reveal the origin of these divergent dislocation accumulation kinetics and hardening rates, we performed multi‐scale microscopy starting with the characterization of dislocation structures. Figure 2 presents TEM micrographs of the as‐solution‐heat‐treated and peak‐aged alloys at different plastic strain levels, highlighting their different deformation modes. In the as‐solution‐treated alloy (ε = 2%, Figure 2a,b), the microstructure is characterized by a relatively homogeneous distribution of dislocations, including numerous dislocation loops, without evidence of planar clustering or strain localization. As the strain increases to 10% (Figure 2c), the dislocation density increases substantially, evolving into dislocation tangles and a cell‐like substructure with an average diameter of 200 nm. The cellular microstructure evolves through the activation of cross‐slip systems, which reduce local shear localization and enable stress relaxation. This process thus facilitates multiplication‐relaxation‐balanced forest dislocation hardening, underpinning the sustained strain hardening and uniform plastic flow across the grain.

In contrast, the peak‐aged alloy exhibits a transition toward localized deformation. Even at an initial strain of 2% (Figure 2d,e), pronounced planar slip bands are observed traversing the grain interiors, often terminating in intense dislocation pile‐ups at grain boundaries (Figure S2). It is important to note that the dislocation activity is confined within these narrow slip bands, without any significant cross‐slip relaxation. This leads to extreme dislocation entanglement and accumulation instead of uniform dislocation patterns. As the strain increases to 10% (Figure 2f), TEM contrast reveals pronounced variations that are a direct signature of severe local misorientation and crystallographic rotation. This phenomenon is further confirmed by the EBSD characterization shown in Figure S3, which displays significant orientation gradients and points of intense stress concentration localized along the active slip bands. This collective evidence demonstrates that the extreme accumulation of dislocations within slip bands leads to lattice rotation within the grain interior. At a strain of 10%, the interaction between dense dislocation tangles and the precipitate becomes intense, triggering structural and chemical changes. Figure S4a shows dislocation shearing of fine precipitates, while Figure S4b identifies multiple pinning points where moving dislocations are obstructed by coarse precipitates. The combined evidence of well‐defined slip bands, localized dislocation tangling, and lattice twisting indicates a severe deformation‐induced destabilization of the peak‐aged microstructure.

**FIGURE 3:**
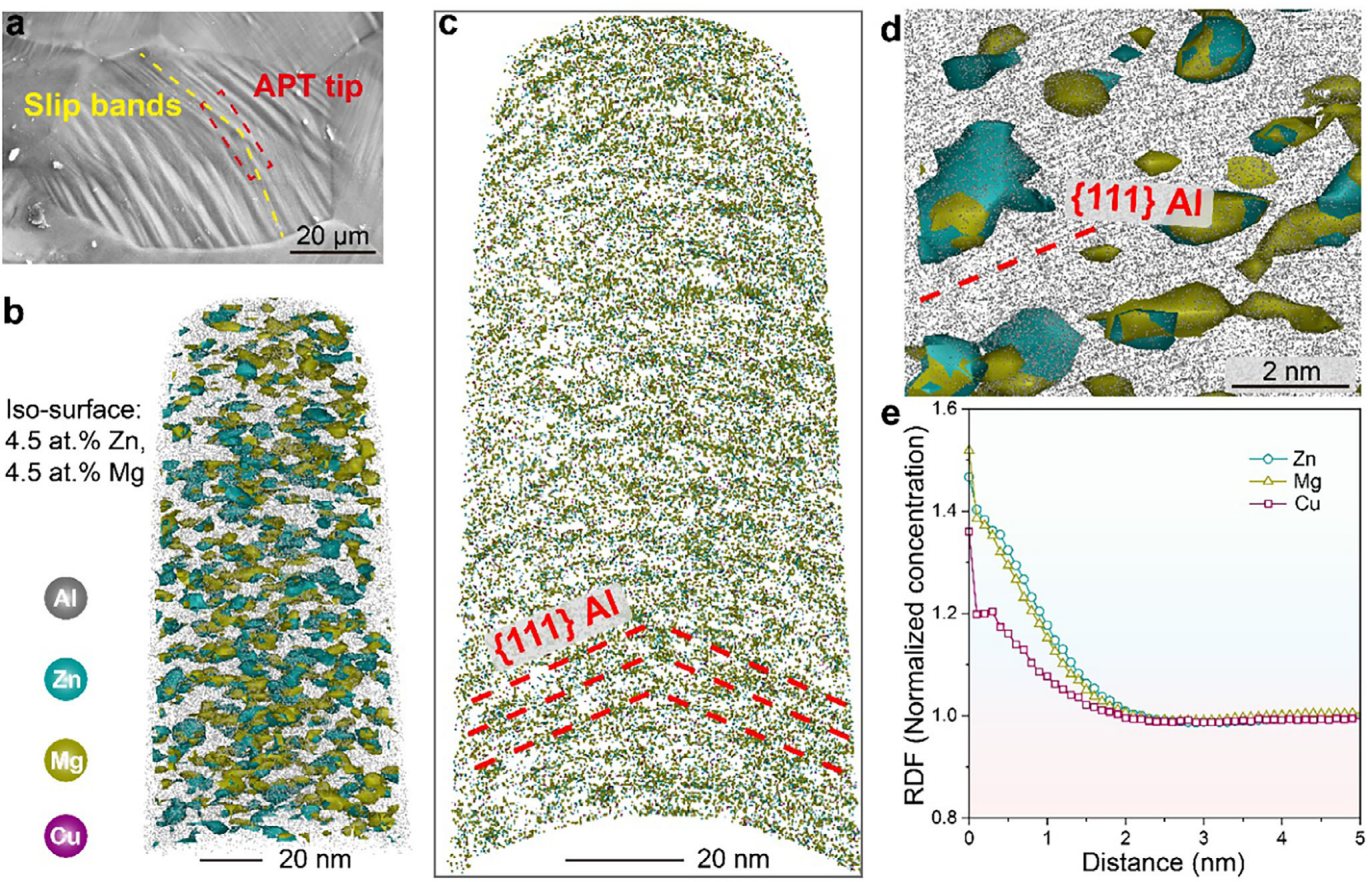
Deformation‐induced solute clustering in the solution‐treated Al–Zn–Mg–Cu alloy after fracture. (a) SEM image showing a representative deformation band in the solution‐treated alloy, with the APT lift‐out location indicated. (b) Three‐dimensional APT reconstruction showing Zn‐ and Mg‐rich clusters after deformation (iso‐surfaces at 4.5 at.% Zn and 4.5 at.% Mg). (c) Atom map revealing the spatial organization of solute clusters, which align along parallel planar features. (d) The close‐up view highlights the preferential alignment of solute clusters along {111} Al planes (iso‐surfaces at 4.5 at.% Zn and 4.5 at.% Mg). (e) RDF curves for Zn, Mg and Cu.

Atom probe tomography was performed on the deformed solution‐treated alloy (Figure 3a) to characterize solute‐dislocation interactions. While the alloy is initially in a supersaturated solid‐solution state prior to deformation, the reconstructed atom maps reveal a distinct strain‐driven clustering effect after deformation. As shown in Figure 3b, nanoscale Zn‐ and Mg‐rich clusters are clearly resolved, as delineated by the iso‐surface with 4.5 at.% Zn and 4.5 at.% Mg. Beyond their emergence, the APT reconstructions, viewed along the {111} plane orientation of the Al matrix (Figure 3c), reveal that the (Zn, Mg)‐rich clusters are preferentially aligned along parallel planar features associated with the {111} slip planes, i.e. the primary slip planes during plastic deformation. A magnified view (Figure 3d) further shows that these clusters are arranged along the {111} planes of the Al matrix. This spatial correlation indicates that the mobile dislocations promote directed solute accumulation, i.e. they act as kinetic vehicles that transport and segregate solute atoms onto their slip planes during plastic flow. This non‐random, dislocation‐promoted solute dispersion is quantitatively validated by the radial distribution function (RDF) analysis for Zn, Mg, and Cu (Figure 3e). In the initial solution‐treated state prior to deformation, the RDF curves for these elements show a statistically near‐homogeneous and random solute distribution (Figure S5). In contrast, the post‐deformation RDF correlations for all three elements exhibit pronounced peaks at short interatomic distances, with the localized enrichment reaching approximately 1.5 times the bulk‐normalized concentration. This deviation from the random baseline confirms the formation of solute clusters. These findings demonstrate that plastic deformation alone is sufficient to trigger dynamic solute clustering, creating a self‐amplifying, strain‐induced strengthening effect that alters the material's local chemical environment encountered by the ensuing glissile dislocations [11].

**FIGURE 4:**
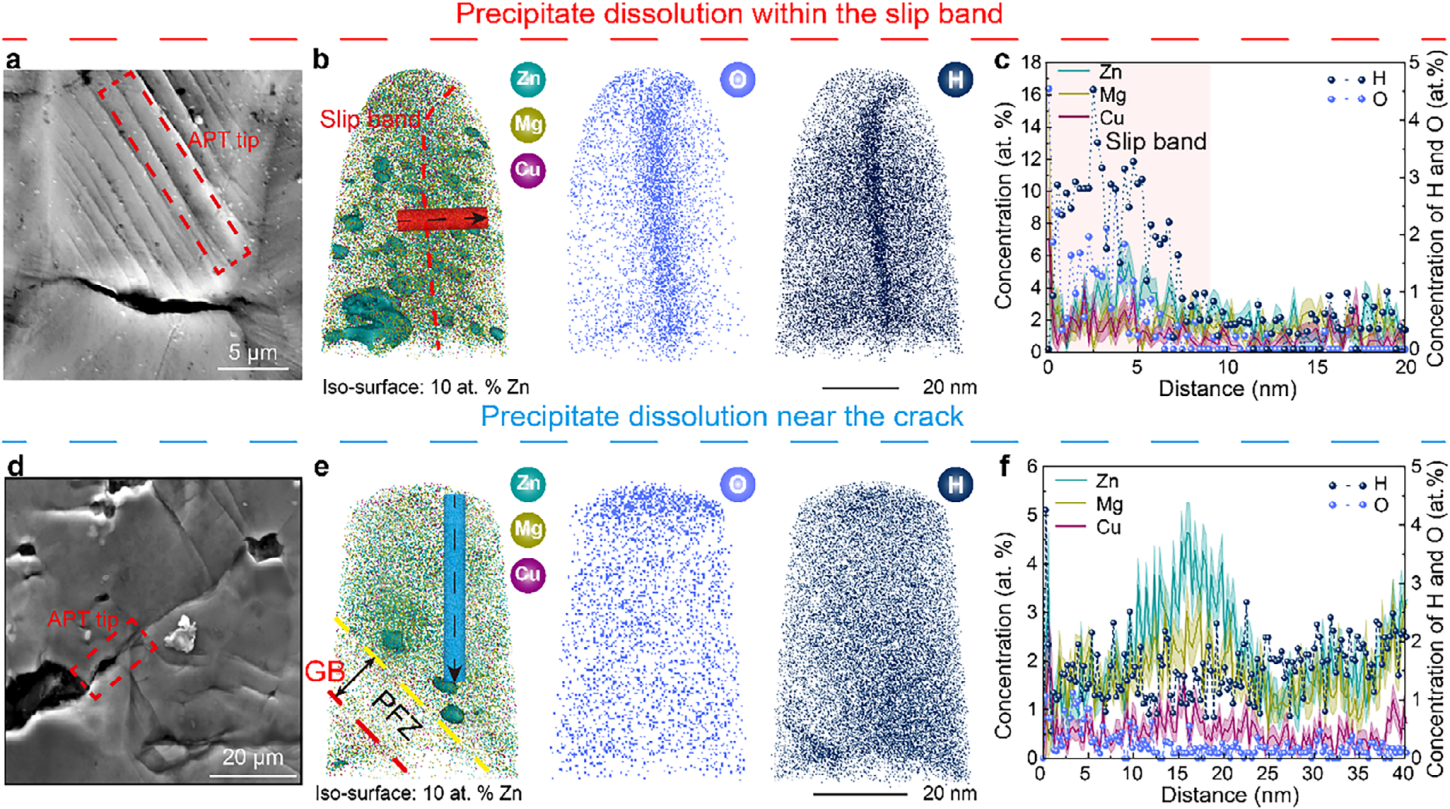
Strain‐induced phase reversion and environmental ingress. (a, d) SEM images showing sampling locations for APT analysis at an active slip band (a) and a through‐thickness crack tip (d). (b) 3D APT reconstructions of the slip band with 10 at.% Zn iso‐surfaces revealing the precipitates and O (blue) and H (dark blue) atom maps. (c) 1D concentration profiles across the slip band in b, quantifying solute release (Zn, Mg, Cu) and environmental enrichment within the slip bands. (e) 3D APT reconstruction of the crack tip near a grain boundary (GB) and precipitate‐free zone (PFZ). (f) 1D concentration profile along the path in e, confirming a nearly twofold increase in solute concentration at the crack‐affected apex relative to the matrix.

The post‐deformation chemical state of the peak‐aged alloy was characterized using APT Figure 4. Analysis was first focused on a region containing active slip bands (Figure 4a). 3D reconstructions and 10 at.% Zn iso‐surfaces (Figure 4b) reveal a localized chemical transition, where the (Mg, Zn)_2_‐rich precipitates undergo complete dissolution confined to the {111} slip planes. This is due to intensive dislocation shearing, where the adjacent matrix retains its initial peak‐aged microstructure populated with nanoscale precipitates. One‐dimensional (1D) concentration profiles across these slip bands (Figure 4c) quantitatively confirm this dissolution, evidenced by a localized increase in solutes reaching twice the bulk concentration, which represents a solute‐release zone from the sheared precipitates. This microstructure contrast between the solute‐dissolved slip bands and the matrix highlights the extreme heterogeneity of the plastic deformation process. Notably, both the atom maps and the concentration profile reveal that O and H are enriched within these precipitate‐dissolved {111} slip planes, rather than being randomly distributed throughout the matrix. This localized enrichment suggests a strong interaction between dynamic dislocation activity and the ingress of environmental species along the slip bands.

Further characterization of the crack tip (Figure 4d) reveals the cumulative consequence of this chemical evolution. The APT analysis presented in Figure 4e captures a volume within the fracture‐affected zone where the initial high‐density precipitates have undergone complete dissolution. This region contains a grain boundary and its associated precipitate‐free zone, as confirmed by the crystallographic features shown in Figure S6. In contrast to the solute‐depleted PFZ [21], the surrounding heavily deformed volume is characterized by a massive solute release resulting from shear‐driven precipitate dissolution. The 1D concentration profile (Figure 4f) confirms this redistribution, with solute levels at the crack‐affected apex reaching approximately twice the nominal matrix concentration. Furthermore, the analysis of H and O reveals a concentration gradient, with the highest levels localized at the specimen apex, corresponding to the high stress at the tip surface. These findings demonstrate the concurrent occurrence of localized strain, precipitate dissolution, and environmental ingress at the site of failure.

**FIGURE 5:**
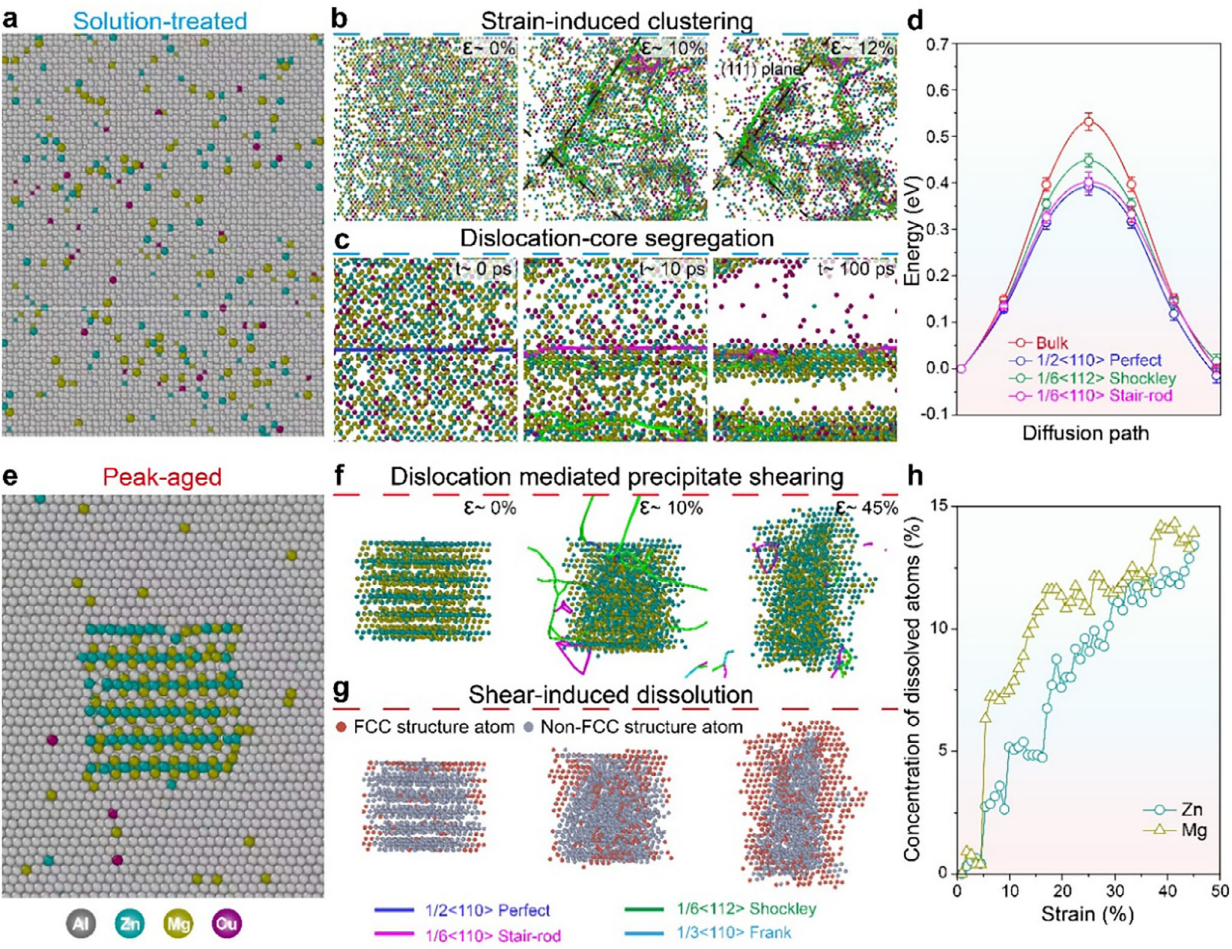
Theoretical analysis of strain‐induced chemical redistribution. (a–c) Solution‐treated alloy: solute clustering. (a), Initial supercell with random solute distribution. (b) MC‐MD simulations of solute atoms and dislocations (colored lines) with strain during precipitation simulation. (c) Static MC‐MD simulation (Al atoms are removed for clarity) showing solute segregation to a 1/2<110> dislocation core, triggering dissociation into Shockley partials. (d), Migration energy barrier calculated via NEB simulations. (e–h) Peak‐aged alloy: shear‐induced dissolution. (e) Initial supercell with an ordered precipitate. (f) MD snapshots of precipitate shearing and elongation. (g) Interval Common Neighbor Analysis showing phase reversion (FCC structure in red; non‐FCC in gray). (h) Concentration of dissolved Zn and Mg atoms within the precipitate as a function of strain.

To further elucidate the atomistic mechanisms governing the observed microstructural instability, we conducted hybrid Monte Carlo‐Molecular Dynamics (MC‐MD) simulations and MD simulations utilizing a NEP‐89 all‐element neural network potential (see Methods). This potential, pre‐trained on multiple datasets with diverse structures and chemistry, was fine‐tuned for the Al‐Zn‐Mg‐Cu system using a reference dataset generated by Marchand et al. [22, 23, 24]. This reference dataset was calculated via density functional theory (DFT) as implemented in Quantum Espresso employing the GGA‐PBE exchange‐correlation functional and PAW pseudopotentials [22]. For both initial states, uniaxial tensile strain in our simulations was applied along the [001] direction at 300 K. In the solution‐treated alloy (Figure 5a,b), deformation triggers a spatial redistribution of solutes, where Mg, Zn, and Cu preferentially segregate to and aggregate along moving dislocation lines. As the strain increases, the rapid increase of dislocations creates a diffusion network that effectively transports solutes and results in the formation of solute‐enriched clusters aligned parallel to the {111} matrix planes. This clustering process is driven by dislocation‐core segregation and dislocation‐promoted pipe diffusion. Static MC‐MD simulation of a 1/2<110> full edge dislocation (Figure 5c) reveals that solutes preferentially segregate to the dislocation core. The local solute enrichment further triggers the dissociation of perfect dislocations into 1/6<112> Shockley partials, driven by energy reduction associated with solute‐dislocation interaction. Nudged Elastic Band (NEB) calculations for solute‐vacancy exchange reveal that the diffusion barrier in bulk Al is 0.53 eV, which decreases to 0.39 eV for a perfect dislocation and about 0.45 eV for Shockley and Stair‐rod partial dislocations. The lower energy barriers confirm that dislocation cores attract solutes and provide high‐diffusivity networks for them, facilitating solute diffusion and the subsequent formation of solute clusters or precipitate dissolution in the peak‐aged state during deformation.

In the peak‐aged state, successive dislocation shearing destabilizes the (Mg, Zn)‐rich precipitates, leading to their gradual dissolution (Figure 5e–h). Atomistic snapshots (Figure 5f) reveal that the precipitates initially undergo morphological elongation along the shearing direction, which increases the interfacial area and provides more sites for solute‐vacancy exchange. This is followed by the preferential displacement of solutes from the precipitate interfaces and their diffusion back into the Al matrix via enhanced diffusion along dislocations. Complementary phase identification via Interval Common Neighbor Analysis (i‐CNA) (Figure 5g) confirms the structural transition from the ordered precipitate lattice to a disordered FCC structure (red‐labeled), identifying a locally supersaturated solid solution. The dissolution kinetics (Figure 5h) further reveal that Mg atoms dissolve at a higher rate than Zn, suggesting that the chemical stability of the Mg sub‐lattice is more sensitive to dislocation‐induced precipitate dissolution. These computational findings provide a qualitative mechanistic understanding for rationalizing our experimental observations of strain‐induced dissolution within localized slip bands and along the crack surface, confirming that the macro‐scale fracture is driven by an atomistic‐scale mechano‐chemical instability.

**FIGURE 6:**
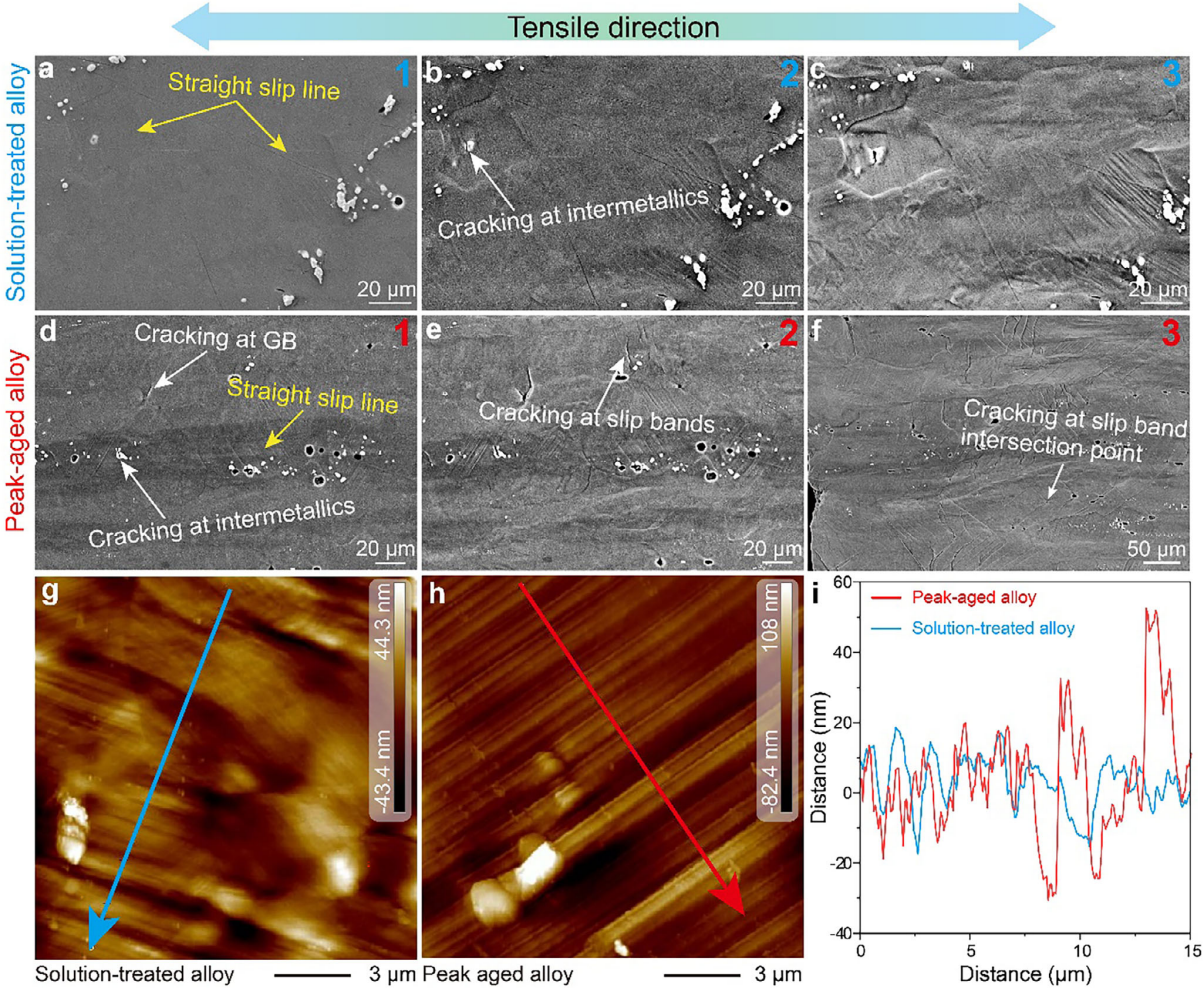
In situ characterization of strain localization and fracture evolution in solution‐treated and peak‐aged Al–Zn–Mg–Cu alloys. (a–f) In situ SEM images of the solution‐treated (a–c) and peak‐aged (d–f) alloys at corresponding strain stages (1–3) marked in Figure 1. (g, h) AFM topographic maps of the slip bands for the solution‐treated alloy (g) and peak‐aged alloy post‐fracture (h). (i) AFM depth profile showing the quantitative analysis of the surface heights for both states.

While the atomistic simulations and chemical mapping provide a mechanistic basis for the observed mechano‐chemical instabilities, it is essential to establish how these nanoscale events manifest as ductility loss at the mesoscale. To bridge this scale gap, Figure 6 displays in situ SEM imaging taken during tensile deformation, alongside nanoscopic AFM topographic mapping, enabling direct visualization of strain localization and crack evolution. The specific deformation stages for the SEM observations are denoted by markers (1, 2, and 3) on the engineering stress–strain curves in Figure 1g. In the solution‐treated alloy (Figure 6a–c), the surface evolution is characterized by spatial homogeneity. At 5% strain (strain level 1), only diffuse and fine slip traces are visible, which remain uniformly distributed across the grains as deformation proceeds to 10% (strain level 2). Throughout these intermediate stages, the sample surface remains free of microcracks, preserving its structural integrity even at strains where the peak‐aged alloy had already failed. Microcracks only emerge during the final stages of deformation beyond a strain of 18% (strain level 3), primarily nucleating at intermetallic inclusion sites. These cracks remain isolated and exhibit no preferential orientation or propagation path along the slip bands. This sequence reveals that the solution‐treated microstructure effectively homogenizes plastic strain and suppresses early crack formation, resulting in a delayed, non‐localized fracture mode.

Conversely, the peak‐aged alloy exhibits sharp and well‐defined slip lines at the early onset of plastic flow. Notably, microscopic cracks are already observed at 5% strain, primarily nucleating at the sites of intense slip bands, grain boundaries, and intermetallic particles. The initiation of microcracks aligns with the sharp drop of the α value observed in the diffraction data (Figure 1i), suggesting that microcracking is linked to the exhaustion of work hardening capacity. As deformation proceeds to 10% strain (strain level 2), these microcracks multiply and propagate along slip planes toward the fracture stage. Post‐fracture analysis (Figure S7) further identifies pronounced, localized slip bands and intense slip steps, confirming that severe deformation localization renders the microstructure incapable of relaxing extreme stress concentration. This forces cracks to propagate along these stress‐localized channels, ultimately leading to catastrophic failure.

To quantify the degree of deformation homogeneity and the intensity of these strain‐localized slip bands observed in SEM, AFM was employed to map the surface step heights within the slip bands of the fractured samples. In the solution‐treated alloy (Figure 6g), plastic strain is distributed uniformly across the lattice. The corresponding surface height profile (Figure 6i) reveals topographic fluctuations that are confined within a 10 nm range. In contrast, the peak‐aged alloy (Figure 6h) exhibits pronounced topographic steps where the corresponding profile shows discrete surface heights exceeding 40 nm and peak‐to‐valley heights approaching 80 nm. This nearly eight‐fold increase in step height serves as a quantitative signature of significant strain localization and the intense stress concentrations during deformation of the peak‐aged alloy.

## 3 Discussion and Conclusion

The present results demonstrate that the mechanical response and eventual failure of Al–Zn–Mg–Cu alloys are governed by the microstructure evolution arising from the solute/precipitates and dislocation interactions during deformation. In the solution‐treated alloy, a sustained hardening capacity (∆σ = 180 MPa) and high ductility of 24% are achieved, reflected by a homogeneous surface topography and a uniform dislocation substructure. The origin of this superior work hardening and stability lies in the formation of (Mg, Zn)‐rich clusters during deformation. This process is driven by the preferential segregation of solutes to dislocation cores and the kinetically facilitated pipe diffusion along the dislocation lines. The latter effect is enabled by a lower migration barrier along the dislocations (0.39 eV) compared to the bulk value (0.53 eV) [25]. These formed (Mg, Zn)‐rich clusters create localized chemical barriers that elevate lattice friction, thereby sustaining a high work‐hardening rate up to 120% higher than that of the peak‐aged state. The persistent hardening forces the activation of cross‐slip systems and effectively suppresses catastrophic strain localization, preventing early failure.

In contrast, the peak‐aged alloy transitions from high initial strength to dynamic mechano‐chemical destabilization shortly after yielding [10, 26, 27]. In situ SEM identifies a strain of 5%, beyond which the alloy loses its plastic stability due to intense, planar slip localization. TEM observations confirm that dislocation activity is confined within {111} slip bands, which drives localized precipitate dissolution. This dissolution process is confirmed by our APT results, which show solute enrichment within the shear zones reaching twice the bulk composition. MD simulations provide a mechanistic understanding of this dissolution process, where gliding dislocations shear through (Mg, Zn)‐rich precipitates and induce morphological elongation along the shearing direction. This structural evolution increases the interfacial area, which facilitates the detachment of solutes from the precipitate interfaces and their diffusion back into the surrounding matrix. This mechanical redistribution leads to the gradual dissolution of the precipitates, with Mg atoms being preferentially removed at a higher rate than Zn, likely driven by their higher vacancy binding energy and larger atomic misfit (12% for Mg vs. 2% for Zn) [28]. The preferential dissolution of Mg atoms leaves behind a transient, Zn‐rich core within the remaining sheared precipitates. This localized stoichiometric deviation destabilizes the chemical coordination of the ordered η’ phase, thereby accelerating the dissolution and transformation from an ordered precipitate lattice to a disordered FCC supersaturated solid solution. This shear‐induced dissolution creates precipitate‐depleted soft zones that undergo intense strain localization, which subsequently act as preferential pathways for the late‐stage ingress of environmental species (H and O). This elemental enrichment occurs as a byproduct of this highly localized deformation and severe structural damage, further reducing the lattice cohesive strength and accelerating catastrophic fracture [29].

In summary, the work hardening behavior and ductility of these two microstructural states are governed by the dynamic interplay between plastic strain and chemical redistribution at the atomic scale. While deformation reinforces the solution‐treated matrix via strain‐induced solute clustering, it destabilizes the peak‐aged matrix through shear‐driven precipitate dissolution. This study shifts our understanding of microstructural features, moving from viewing precipitates as static strengthening obstacles to recognizing them as highly dynamic participants in an alloy's mechano‐chemical stability during deformation. Specifically, localized deformation drives shear‐induced precipitate dissolution in peak‐aged alloys, which in turn intensifies strain localization within soft channels and accelerates early failure. Understanding these divergent pathways provides critical mechanistic insights for tailoring phase stability to suppress severe strain localization and guide the future design of damage‐tolerant lightweight materials.

## 4 Methods

### Material Processing and Heat Treatment

The 7xxx Al alloy investigated has a nominal composition of Al–6.22Zn–2.46Mg–2.13Cu–0.16Zr–0.02Fe (wt.%) (equivalent to Al–2.69Zn–2.87Mg–0.95Cu–0.05Zr–0.01Fe in at.%). The as‐cast alloy was homogenized at 475°C for 24 h and subsequently hot‐rolled from 40 mm to 3 mm at 450°C. The hot‐rolled plates were solution heat‐treated at 475°C for 24 h, followed by immediate water quenching. Two microstructural conditions were produced: solution heat‐treated (SHT, 475°C for 24 h) and peak‐aged (120°C for 24 h).

### Microstructural and Chemical Characterization

APT and TEM specimens were fabricated using Xe^+^ focused ion beam system (Thermo Fisher Plasma FIB) operated at 30 kV, followed by a final 5 kV cleaning step to minimize surface damage. A 1 µm‐thick Pt capping layer was deposited via electron‐beam (10 kV, 1.6 nA) to protect the specimen surface prior to lift‐out. Standard lift‐out and sharpening procedures were employed using Si microtip arrays for APT and Mo grids for TEM.

APT experiments were conducted immediately following specimen preparation using a LEAP 5000XR system. Analysis was operated in voltage‐pulsing mode at a cryogenic temperature of 30 K, with a pulse fraction of 20% and a repetition rate of 250 kHz. Data reconstruction and quantitative analysis were executed using AP Suite 6.1, with spatial calibration based on detected crystallographic poles. At least three APT datasets were acquired for each condition to ensure statistical reproducibility.

SEM and EBSD analyses were performed using a Zeiss Gemini 500 microscope operating at 20 kV. TEM and STEM analyses were performed using an aberration‐corrected Thermo Fisher Titan Themis microscope operated at 300 kV. The aberration‐corrected probe provided a convergence semi‐angle of 24 mrad and a spatial resolution better than 0.1 nm. HAADF ‐STEM images were acquired using detectors with collection angles of 73–200 mrad.

### In Situ Deformation and Mechanical Testing

Uniaxial tensile tests were performed at room temperature using an INSTRON‐5966 universal testing machine at an initial strain rate of 1 × 10^−3^ s^−1^. Dog‐bone specimens with a gauge length of 20 mm and a cross‐section of 1.0 × 2.5 mm^2^ were used (Figure 1g). Strain was measured using a clip‐on extensometer.

The in situ procedure was performed using a Kammrath‐Weiss tensile stage (maximum loading of 5 kN) integrated with in a CIQTEK SEM 4000 microscope operated at 20 kV. A Gatan microtension stage was utilized at a strain rate of 1 × 10^−3^ s^−^^1^. Dog‐bone samples (1 × 1 mm^2^ cross‐section, 3 mm gauge length) were used (Figure 6).

### In Situ Synchrotron High‐Energy X‐Ray Diffraction

High‐energy XRD experiments were performed at Beamline P21.2 of the DESY Synchrotron Radiation Facility. Dog‐bone samples (1 × 1 mm^2^ cross‐section, 3 mm gauge length) were used (Figure 1i). The experiments employed 52 keV x‐rays (λ = 0.0238 nm) and a 0.5 × 0.5 mm^2^ beam size in transmission geometry. Two‐dimensional diffractograms were recorded every 10 s using a Perkin‐Elmer detector while applying tensile loading with a micro tension tester at a strain rate of 1 × 10^−3^ s^−1^.

Dislocation densities were quantitatively determined using the Williamson–Hall method [19, 30, 31]. The broadening of the diffraction reflection is mainly attributed to the average grain size (D) broadening and strain (ε) broadening.

$$\mathrm{Bcos}\;\theta_{\mathrm{hkl}}=K\lambda /D+\varepsilon \sin\theta_{\mathrm{hkl}}s$$

where B is the actual high‐energy XRD peak width. ***K*** ∼ 0.9 is a constant; ***λ*** is the wavelength of the diffraction. ***D*** is the average grain size ***θ***_***hkl***_ is the Bragg angle of the characteristic peak. The values of ***D*** and ***ε*** can be calculated from the intercept and slope of the linearly fitted lines of ***Bcosθ***_***hkl***_ as a function of ***sinθ***_***hkl***_. Then, the dislocation density of the peak‐aged and solution‐treated alloys can be calculated from the following equation:

$$\rho =2\sqrt{3}\varepsilon /D\left|b\right|$$

where ***b*** = $\sqrt{2}\;a/2$ is the magnitude of the Burgers vector of the FCC phase. a = 4.4045 Å is the lattice constant of 7xxx Al alloy.

The Taylor α can be determined by the following equation [20]:

$$\sigma_{1}-\;\sigma_{2}=M\alpha Gb\left(\sqrt{\rho_{1}}-\sqrt{\rho_{2}}\right)$$

where the Taylor factor M is 3.06. G = 26 GPa is the shear modulus of 7xxx Al alloy. The values of* *α can be calculated by the intercept and slope the linearly fitted lines of σ_1_ − σ_2_ as a function of $\sqrt{\rho_{1}}-\sqrt{\rho_{2}}$.

### Atomistic Simulations

The atomistic simulations in this study were based on the Graphics Processing Units Molecular Dynamics (GPUMD) package [23], utilizing the NEP‐89 all‐element neural network potential [24]. The NEP potential was adopted due to its high computational efficiency. To accurately describe the Al‐Zn‐Mg‐Cu alloy system, we fine‐tuned the NEP‐89 potential for 100,000 steps using the DFT dataset generated by Marchand et al. [22]. The fine‐tuned potential achieved a root‐mean‐square error (RMSE) of 0.014 eV/atom for energy and 71 meV/Å for force on the test set, demonstrating performance comparable to the potential trained by Marchand et al., but with much higher computational efficiency that meets our expensive atomistic simulations for large atomic supercells and extended time scales.

To simulate the process of dislocation‐promoted precipitate formation, a hybrid MC‐MD method was employed [32]: after every 150 steps of molecular dynamics (MD), 1000 steps of Monte Carlo (MC) sampling were performed. The MC process was conducted at 300 K, while the MD simulations utilized an Isothermal‐isobaric (NPT) ensemble at 300 K. Tensile strain was applied along the [001] direction at a strain rate of 5 × 10^8^ s^−1^ to a total strain of 15%. The alloy model contains approximately 180 000 atoms, as shown in Figure 5a. Furthermore, a separate model containing about 40 000 atoms and two 1/2<110> perfect dislocations was constructed to investigate the solute segregation effect at dislocations. The same MC‐MD simulation parameters were applied to this model, with the exception of the tensile strain.

To investigate the dislocation‐induced dissolution of the secondary phase, conventional MD simulations were performed. These MD simulations were conducted in an NPT ensemble at 300 K, with tensile loading applied along the [001] direction at a strain rate of 1.5 × 10^7^ s^−1^ to a total strain of 45%. The model contained about 84 000 atoms, consisting of a hexagonal Al_4_Mg_2_Zn_3_ precipitate with a side length of 4 nm embedded in a cubic alloy matrix with a side length of 11 nm, as illustrated in Figure 5e. The choice of the model precipitate phase is based on our experimental APT measurements (Figure 1), which revealed a chemical composition close to Al_4_Mg_2_Zn_3_. Density functional theory (DFT) calculations confirm that this configuration represents the energetically most stable structure for the η′ phase,  which is also in good agreement with HAADF‐STEM Z‐contrast observations of the 7xxx Al alloy [33]. The embedded η′ precipitate was constructed with the established orientation relationship with the Al matrix, [10‐10]η_′_ // [110]_Al_, (0001)_η′_ // (111)_Al_. Phase identification was conducted using the i‐CNA approach [34].

To investigate the dislocation‐induced diffusion effect, we calculated the diffusion energy barriers for the bulk, 1/2<110> perfect dislocation core, 1/6<112> Shockley and 1/6<110> Stair‐rod dislocation core using a dislocation‐containing model with approximately 5 000 atoms. For the Al‐Zn‐Mg‐Cu alloy, the alloy atoms were randomly reassigned before each NEB simulation. A solute atom (Mg, Cu, or Zn) at the dislocation core was randomly selected as the mobile species, and one of its four nearest neighbors was chosen as the target. After removing the target atom to create a vacancy, the mobile atom was moved to the target position. Both the initial and final structures were relaxed for cell and atomic positions with a force tolerance of 0.01 eV/Å. The diffusion path was calculated using the dynamic NEB (DyNEB) [35, 36] method with 5 intermediate images, converged to a force tolerance of 0.05 eV/Å. Statistical distributions of diffusion energy barriers were obtained through 40 repetitions, and the results are shown in Figure 5d, with error bars representing the standard deviation of the energy distribution.

## Author Contributions

H.Z. conceived the research, led the overall investigations, and wrote the manuscript; L.L. coordinated the TEM characterization, analyzed the in situ x‐ray and AFM data, and contributed to the manuscript preparation; S.W. conducted the synchrotron x‐ray diffraction measurements; J.Q. and X.Z. performed the theoretical simulations; J.X. and D.X. carried out the in situ SEM measurements. All authors contributed to the scientific discussion and revised the manuscript.

## Conflicts of Interest

The authors declare no conflicts of interest.

## Supporting information




**Supporting File**: advs77786‐sup‐0001‐SuppMat.docx.

## Data Availability

The data that support the findings of this study are available in the supplementary material of this article.

## References

[1] I. Polmear , D. StJohn , J.‐F. Nie , and M. Qian , Light Alloys: Metallurgy of the Light Metals (Butterworth‐Heinemann, 2017).

[2] P. A. Rometsch , Y. Zhang , and S. Knight , “Heat Treatment of 7xxx Series Aluminium Alloys—Some Recent Developments,” Transactions of Nonferrous Metals Society of China 24, no. 7 (2014): 2003–2017, 10.1016/s1003-6326(14)63306-9.

[3] E. A. Starke and J. T. Staley , “Application of Modern Aluminum Alloys to Aircraft,” Progress in Aerospace Sciences 32, no. 2 (1996): 131–172, 10.1016/0376-0421(95)00004-6.

[4] H. Zhao , B. Gault , D. Ponge , D. Raabe , and F. De Geuser , “Parameter Free Quantitative Analysis of Atom Probe Data by Correlation Functions: Application to the Precipitation in Al‐Zn‐Mg‐Cu,” Scripta Materialia 154 (2018): 106–110, 10.1016/j.scriptamat.2018.05.024.

[5] G. Sha and A. Cerezo , “Early‐Stage Precipitation in Al–Zn–Mg–Cu alloy (7050),” Acta Materialia 52, no. 15 (2004): 4503–4516, 10.1016/j.actamat.2004.06.025.

[6] T. Marlaud , A. Deschamps , F. Bley , W. Lefebvre , and B. Baroux , “Influence of Alloy Composition and Heat Treatment on Precipitate Composition in Al–Zn–Mg–Cu Alloys,” Acta Materialia 58, no. 1 (2010): 248–260, 10.1016/j.actamat.2009.09.003.

[7] A. K. Vasudevan and R. Doherty , “Grain Boundary Ductile Fracture in Precipitation Hardened Aluminum Alloys,” Acta Metallurgica 35, no. 6 (1987): 1193–1219, 10.1016/0001-6160(87)90001-0.

[8] D. Dumont , A. Deschamps , and Y. Brechet , “On the Relationship Between Microstructure, Strength and Toughness in AA7050 Aluminum Alloy,” Materials Science and Engineering: A 356, no. 1‐2 (2003): 326–336, 10.1016/s0921-5093(03)00145-x.

[9] M. J. Starink and S. C. Wang , “A Model for the Yield Strength of Overaged Al–Zn–Mg–Cu Alloys,” Acta Materialia 51, no. 17 (2003): 5131–5150, 10.1016/s1359-6454(03)00363-x.

[10] Y. Brechet , F. Louchet , C. Marchionni , and J. L. Verger‐Gaugry , “Experimental (TEM and STEM) Investigation and Theoretical Approach to the Fatigue‐Induced Dissolution of δ′ Precipitates in a 2.5 wt% Al‐Li Alloy,” Philosophical Magazine A 56, no. 3 (1987): 353–366, 10.1080/01418618708214391.

[11] W. Sun , Y. Zhu , R. Marceau , et al., “Precipitation Strengthening of Aluminum Alloys by Room‐Temperature Cyclic Plasticity,” Science 363, no. 6430 (2019): 972–975, 10.1126/science.aav7086. 30819960

[12] G. Fribourg , Y. Bréchet , A. Deschamps , and A. Simar , “Microstructure‐Based Modelling of Isotropic and Kinematic Strain Hardening in a Precipitation‐Hardened Aluminium Alloy,” Acta Materialia 59, no. 9 (2011): 3621–3635, 10.1016/j.actamat.2011.02.035.

[13] W. Muhammad , R. K. Sabat , A. P. Brahme , et al., “Deformation Banding in a Precipitation Hardened Aluminum Alloy During Simple Shear Deformation,” Scripta Materialia 162 (2019): 300–305, 10.1016/j.scriptamat.2018.11.032.

[14] U. F. Kocks and H. Mecking , “Physics and Phenomenology of Strain Hardening: The FCC Case,” Progress in Materials Science 48, no. 3 (2003): 171–273, 10.1016/S0079-6425(02)00003-8.

[15] P. V. Liddicoat , T. Honma , L. T. Stephenson , and S. P. Ringer , “Evolution of Nanostructure During the Early Stages of Ageing in Al‐Zn‐Mg‐Cu Alloys,” Materials Science Forum 519–521 (2006): 555–560, 10.4028/www.scientific.net/MSF.519-521.555.

[16] W. Vogel , M. Wilhelm , and V. Gerold , “Persistent Slip Bands in Fatigued Peak Aged Al Zn Mg Single Crystals—I. Development of Dislocation Microstructure and Change of Precipitation Distribution,” Acta Metallurgica 30, no. 1 (1982): 21–30, 10.1016/0001-6160(82)90040-2.

[17] S. Suresh , A. K. Vasudévan , and P. E. Bretz , “Mechanisms of Slow Fatigue Crack Growth in High Strength Aluminum Alloys: Role of Microstructure and Environment,” Metallurgical Transactions A 15, no. 2 (1984): 369–379, 10.1007/BF02645122.

[18] I. S. Yasnikov , A. Vinogradov , and Y. Estrin , “Revisiting the Considère Criterion From the Viewpoint of Dislocation Theory Fundamentals,” Scripta Materialia 76 (2014): 37–40, 10.1016/j.scriptamat.2013.12.009.

[19] Z. An , A. Li , S. Mao , et al., “Negative Mixing Enthalpy Solid Solutions Deliver High Strength and Ductility,” Nature 625, no. 7996 (2024): 697–702, 10.1038/s41586-023-06894-9. 38172639

[20] H. Mughrabi , “The α‐Factor in the Taylor Flow‐Stress Law in Monotonic, Cyclic and Quasi‐Stationary Deformations: Dependence on Slip Mode, Dislocation Arrangement and Density,” Current Opinion in Solid State and Materials Science 20 (2016): 411–420, 10.1016/j.cossms.2016.07.001.

[21] H. Zhao , F. De Geuser , A. K. da Silva , et al., “Segregation Assisted Grain Boundary Precipitation in a Model Al‐Zn‐Mg‐Cu Alloy,” Acta Materialia 156 (2018): 318–329, 10.1016/j.actamat.2018.07.003.

[22] D. Marchand and W. Curtin , “Machine Learning for Metallurgy IV: A Neural Network Potential for Al‐Cu‐Mg and Al‐Cu‐Mg‐Zn,” Physical Review Materials 6, no. 5 (2022): 053803, 10.1103/PhysRevMaterials.6.053803.

[23] Z. Fan , W. Chen , V. Vierimaa , and A. Harju , “Efficient Molecular Dynamics Simulations With Many‐Body Potentials on Graphics Processing Units,” Computer Physics Communications 218 (2017): 10–16, 10.1016/j.cpc.2017.05.003.

[24] T. Liang , K. Xu , E. Lindgren , et al., “NEP89: Universal Neuroevolution Potential for Inorganic and Organic Materials Across 89 Elements,” Nature Computational Science 6, (2026): 789–801, 10.1038/s43588-026-01009-6. 42420553

[25] A. Deschamps , G. Fribourg , Y. Bréchet , J. L. Chemin , and C. R. Hutchinson , “In Situ Evaluation of Dynamic Precipitation During Plastic Straining of an Al–Zn–Mg–Cu Alloy,” Acta Materialia 60, no. 5 (2012): 1905–1916, 10.1016/j.actamat.2012.01.002.

[26] C. R. Hutchinson , P. T. Loo , T. J. Bastow , A. J. Hill , and J. da Costa Teixeira , “Quantifying the Strain‐Induced Dissolution of Precipitates in Al Alloy Microstructures Using Nuclear Magnetic Resonance,” Acta Materialia 57, no. 19 (2009): 5645–5653, 10.1016/j.actamat.2009.07.060.

[27] D. Steiner , R. Beddoe , V. Gerold , G. Kostorz , and R. Schmelczer , “Particle Dissolution During Fatigue Softening of Age Hardened Cu Co Single Crystals,” Scripta Metallurgica 17, no. 6 (1983): 733–736, 10.1016/0036-9748(83)90483-0.

[28] C. Wolverton , “Solute–Vacancy Binding in Aluminum,” Acta Materialia 55, no. 17 (2007): 5867–5872, 10.1016/j.actamat.2007.06.039.

[29] A. A. Csontos and E. A. Starke , “The Effect of Inhomogeneous Plastic Deformation on the Ductility and Fracture Behavior of Age Hardenable Aluminum Alloys,” International Journal of Plasticity 21, no. 6 (2005): 1097–1118, 10.1016/j.ijplas.2004.03.003.

[30] G. Dini , R. Ueji , A. Najafizadeh , and S. Monir‐Vaghefi , “Flow Stress Analysis of TWIP Steel via the XRD Measurement of Dislocation Density,” Materials Science and Engineering: A 527, no. 10‐11 (2010): 2759–2763, 10.1016/j.msea.2010.01.033.

[31] R. Smallman and K. Westmacott , “Stacking Faults in Face‐Centred Cubic Metals and Alloys,” Philosophical Magazine 2 no. 17 (1957): 669–683, 10.1080/14786435708242709.

[32] K. Song , J. Liu , S. Chen , Z. Fan , Y. Su , and P. Qian , “Solute Segregation in Polycrystalline Aluminum From Hybrid Monte Carlo and Molecular Dynamics Simulations With a Unified Neuroevolution Potential,” Materials Genome Engineering Advances 4 (2026): e70049, 10.1002/mgea.70049.

[33] F. Cao , J. Zheng , Y. Jiang , B. Chen , Y. Wang , and T. Hu , “Experimental and DFT Characterization of η′ Nano‐Phase and its Interfaces in Al Zn Mg Cu Alloys,” Acta Materialia 164 (2019): 207–219, 10.1016/j.actamat.2018.10.045.

[34] P. M. Larsen , “Revisiting the Common Neighbour Analysis and the Centrosymmetry Parameter,” arXiv (2020), 10.48550/arXiv.2003.08879.

[35] A. Hjorth Larsen , J. Jørgen Mortensen , J. Blomqvist , et al., “The Atomic Simulation Environment—a Python Library for Working With Atoms,” Journal of Physics: Condensed Matter 29, no. 27 (2017): 273002, 10.1088/1361-648X/aa680e. 28323250

[36] P. Lindgren , G. Kastlunger , and A. A. Peterson , “Scaled and Dynamic Optimizations of Nudged Elastic Bands,” Journal of Chemical Theory and Computation 15, no. 11 (2019): 5787–5793, 10.1021/acs.jctc.9b00633. 31600078

