# Supplementary material for: When Mechanics Rewrites Chemistry: Divergent Pathways of Hardening and Softening in High Strength Al‐Alloys
Source: Adv Sci (Weinh). 2026 Sep 16:e77786. Online ahead of print. doi: 10.1002/advs.77786 (PMC13583222; doi:10.1002/advs.77786)
Supplement: Supplementary file 1 [file ADVS-9999-e77786-s001.docx]

**Supplementary Information for**

**When mechanics rewrites chemistry: Divergent pathways of hardening and softening in high strength Al-alloys**

Huan Zhao^1,*^, Linze Li^1^, Shaolou Wei^2^, Jueyi Qi^3^, Jiewen Xiao^1^, Degang Xie^1^, Xie Zhang^3,*^, Gang Liu^1,*^, Dierk Raabe^2, *^, and Jun Sun^1^

1. State Key Laboratory for Mechanical Behavior of Materials, Xi’an Jiaotong University, Xi’an, P.R. China
2. Max-Planck-Institute for Sustainable Materials, Düsseldorf, Germany
3. School of Materials Science and Engineering, Northwestern Polytechnical University, Xi’an, 710072, P.R. China

Corresponding author: [h.zhao@xjtu.edu.cn](mailto:h.zhao@xjtu.edu.cn); [xie.zhang@nwpu.edu.cn](mailto:xie.zhang@nwpu.edu.cn); [lgsammer@mail.xjtu.edu.cn](mailto:lgsammer@mail.xjtu.edu.cn); d.raabe@[mpi-susmat.de](mailto:d.raabe@mpi-susmat.de)

**Figure S1 Evolution of dislocation density as a function of true strain measured by *in-situ* high-energy synchrotron X-ray diffraction.**

**Figure S2 HAADF-STEM image showing intense dislocation pile-ups at a grain boundary (GB) of the peak aged alloy at the plastic strain of 10%.** The blue dashed line marks the GB.

**Figure S3 Multi-scale characterization of the peak-aged alloy after fracture.** (a) SEM secondary electron image showing the surface morphology near a crack. Pronounced macroscopic slip steps are observed along the {111} planes (yellow dashed lines), terminating at the grain boundary (GB, cyan line). (b) Corresponding EBSD inverse pole figure (IPF) map of the region boxed in (a). Significant color gradients within individual grains and across slip bands provide quantitative evidence of severe local lattice rotation and crystallographic misorientation. (c) Kernel average misorientation (KAM) map highlighting the spatial distribution of local geometrically necessary dislocation (GND) density and strain. The highest KAM values (red regions) are strictly localized along the active slip bands and at the GB interface, confirming the presence of intense stress concentrations.

**Figure S4 STEM analysis of dislocation-precipitate interactions in the peak-aged alloy at 10% strain.** (a) BF-STEM micrograph revealing extensive dislocation shearing of the fine precipitates. (b) DF-STEM micrograph highlighting the pinning effect of coarser precipitates on moving dislocations. Yellow arrows identify multiple pinning points where dislocation lines are effectively obstructed.

**Figure S5 APT analysis of the solution-treated Al–Zn–Mg–Cu alloy.** (a) Three-dimensional APT reconstruction, (b) Radial distribution functions (RDFs) for Zn, Mg and Cu.

**Figure S6 APT reconstruction and corresponding pole figures of the grain boundary region.** (a) Tomographic reconstruction of the entire dataset; (b Pole figures from the grain boundary region in this dataset, with the GB shown by the yellow line.

**Figure S7 SEM characterization of strain localization and slip-band-induced cracking.** (a) SEM showing the global distribution of deformation features near the fracture surface. (b) High-magnification view of the region indicated by the green box, highlighting cracks formed align the slip band. (c) Detailed analysis of localized slip bands, where pronounced macroscopic slip steps are observed along the {111} close-packed planes.
